# Distribution of the Galβ1-4Gal Epitope among Birds: Species-Specific Loss of the Glycan Structure in Chicken and Its Relatives

**DOI:** 10.1371/journal.pone.0059291

**Published:** 2013-03-19

**Authors:** Noriko Suzuki, Daisuke Nawa, Tseng-Hsiung Su, Chia-Wei Lin, Kay-Hooi Khoo, Kazuo Yamamoto

**Affiliations:** 1 Department of Integrated Biosciences, Graduate School of Frontier Sciences, University of Tokyo, Kashiwa, Japan; 2 Institute of Biochemical Sciences, National Taiwan University, Taipei, Taiwan; 3 Institute of Biological Chemistry, Academia Sinica, Nankang, Taipei, Taiwan; Centro de Biología Molecular Severo Ochoa (CSIC-UAM), Spain

## Abstract

The Galβ1-4Gal epitope is rarely found in mammals, and the natural antibody against Galβ1-4Gal is rich in human. In contrast, we have previously demonstrated the presence of Galβ1-4Gal in pigeon and ostrich, and the absence of this epitope in chicken. Here, to further investigate the expression of this glycan among birds, egg white glycoproteins and egg yolk IgG from nine species of birds, namely, chicken, duck, emu, guineafowl, ostrich, peafowl, pigeon, quail, and turkey, were analyzed by western blot using an anti-(Galβ1-4Gal) antibody. The results indicated that some egg white glycoproteins from emu, ostrich, and quail, and heavy chains of IgG from all of the birds, except chicken and quail, were stained with the antibody. The presence of Galβ1-4Gal on *N*-glycans of IgGs from guineafowl, peafowl, and turkey were confirmed by mass spectrometry (MS), MS/MS, and MS^n^ analyses. In quail, the presence of Galβ1-4Gal was confirmed by detecting the activities of UDP-galactose: β-galactoside β1,4-galactosyltransferase (β4GalT(Gal)) in various tissues, and by detecting Galβ1-4Gal by western blotting. In contrast, bamboo partridge, which is a close relative of chicken, did not show any detectable activities of β4GalT(Gal) or Galβ1-4Gal on glycoproteins. Because quail, peafowl, turkey, chicken, and bamboo partridge belong to the same family, i.e., Phasianidae, expression of Galβ1-4Gal was most likely differentiated within this family. Considering that Galβ1-4Gal is also expressed in ostrich, emu, and pigeon, which are phylogenetically distant relatives within modern birds, Galβ1-4Gal expression appears to be widely distributed among birds, but might have been abolished in the ancestors of chicken and bamboo partridge.

## Introduction

Species-specific structures of glycans attached to glycoproteins or glycolipids are often found in nature. While well-conserved glycan structures among species are often crucial for the homeostasis of organisms that synthesizes them, the biological roles of species-specific glycans are not well understood. One of the hypothetical scenarios is that glycans are evolutionally differentiated to gain species-specific communication between self (e.g., hosts) and non-self (e.g., foreign organisms), either for pathogenic or symbiotic relationships [1], [2], [3]. Because all cells in nature are covered with a dense coating of glycans, some structures of glycans are often used as targets that are recognized by carbohydrate-binding proteins expressed on foreign microbes or exotoxins. By changing particular glycan structures on cell surfaces, for example, hosts are able to evade attachments of pathogens mediated by the carbohydrate recognition. Another possible advantage of species-specific glycans is that hosts are able to produce antibodies against carbohydrate antigens on pathogens, when the hosts do not express the same carbohydrate epitopes [4]. It is proposed that anti-carbohydrate antibodies may act as barriers to retrovirus transmission between positive and negative taxa [2], [5].

Although the presence of species-specific glycans are assumed to be important for the biological defense system, relatively little is known about the generation and distribution of glycan diversity in nature [6]. While in mammals, especially in humans and mice, comprehensive studies on the glycan structures, glycosyltransferases, and biological roles of glycans are in progress, only limited information is currently available regarding the distribution of species-specific glycans in non-mammalian vertebrates. However, glycan structures in nature might be the consequence of the gain and loss of abilities to express various structures of glycans, throughout the long history of living organisms [1], [2]. To find out the mechanism underlying the species-specific expression of glycans and to understand the biological significance of such glycans in detail, systematic investigations are necessary in a wide range of animals, not limited to mammals.

We have previously revealed that birds possess different glycan profiles from those of mammals. One of the unique glycans in birds is the Galα1-4Gal epitope found on glycoproteins. When we analyzed egg white glycoproteins from 181 avian species, we showed that Galα1-4Gal on glycoproteins is present in a major lineage of avian species called Neoaves^*^ (including, e.g., pigeon, gull, parrot, and swiftlet), but absent in the other lineages of modern birds, namely, Ratitae (traditionally called Palaeognathae, e.g., ostrich, emu) and Galloanserae (e.g., chicken, duck) [7], [8], [9]. (^*^Nomenclatures for avian classification are based on Sibley *et al.* [9]. In this paper, Neoaves does not include Galloanserae.).

The other unique glycan in birds is the Galβ1-4Gal epitope on glycoproteins, which was originally found in *O*-glycans of salivary gland mucin from Chinese swiftlet [10] and *N*-glycans of IgG from pigeon [11]. While Galα1-4Gal is abundant in both pigeon egg white glycoproteins [12], [13] and IgG [11], the Galβ1-4Gal epitope was not found in pigeon egg white glycoproteins [12], [13]. Unlike Galα1-4Gal, distribution of Galβ1-4Gal in avian species has not been well studied. To address this issue, we recently developed specific antibodies capable of binding to the non-reducing termini of Galβ1-4Gal [14]. Moreover, we have also established a method to detect specific activities of UDP-galactose: β-galactoside β1,4-galactosyltransferase (β4GalT(Gal)^‡^), which is responsible for the production of Galβ1-4Gal epitope on *N*-glycans [15]. (^‡^In this paper, GalTs are conveniently abbreviated as linkageGalT(acceptor substrate) to distinguish their acceptor substrate specificities each other, e.g., UDP-galactose: β-d-galactoside β1,4-galactosyltransferase is designated as β4GalT(Gal).) Based on these assays, we have found that Galβ1-4Gal on glycoproteins was expressed not only in pigeon, but also in ostrich [15]. Since pigeon and ostrich belong to Neoaves and Ratitae, respectively, which are distantly related modern bird taxa, other birds that are phylogenetically closer to these birds also possibly express the Galβ1-4Gal epitope. However, we detected no β4GalT(Gal) activities or Galβ1-4Gal on glycoproteins in various tissues of chicken, which belongs to Galloanserae. Therefore, it remained unclear whether the ability to express Galβ1-4Gal on glycoproteins was acquired independently in pigeon and ostrich, or whether it was inherited from common ancestors of modern birds and was somehow lost in chicken.

In this study, to further investigate the distribution of Galβ1-4Gal in avian species, especially among close relatives to chicken, we first analyzed egg white glycoproteins and egg yolk IgGs, also called IgYs, from nine species of birds. The results suggest that Galβ1-4Gal is expressed in a wider range of avian species than previously recognized, and that the ancestors of chicken and bamboo partridge might have lost the ability to produce Galβ1-4Gal.

## Materials and Methods

### Materials

Adult female Japanese quail, and Chinese bamboo partridge, and eggs from duck and helmet guineafowl, were purchased from Saitama Experimental Animals Supply Co. Eggs from emu, ostrich, Indian peafowl, and wild turkey were purchased from a local farmer in the Ibaraki area. Eggs from chicken and Japanese quail were purchased from local grocery stores in the Kashiwa area. Anti-P_1_ mAb (mouse IgM) was from Gamma Biologicals (Houston, TX). anti-(Galβ1-4Gal) mAb 68 (mouse IgG_1_) were prepared as described previously [14]. All other materials used in this study were the same as described previously [15]. All animal experimentations were conducted in accordance with the Guidelines for Proper Conduct of Animal Experiments (Science Council of Japan), and all protocols were approved by the review boards of Animal Experiments Committee of the University of Tokyo (Permit Number: 07-C-9).

### Standard Procedures

Protein concentrations were measured by the BCA assay using the BCA Protein Assay Reagent Kit (Pierce, Rockford, IL), or by the Bradford assay using Coomassie Plus Reagent (Pierce).

### Isolation of Egg Yolk IgG

Lyophilized egg yolks (4 g) were dissolved with 100 ml of distilled water, and centrifuged to remove insoluble materials. Egg yolk IgG was isolated with the Eggcellent™ Chicken IgY Purification Kit (Pierce), and further purified by gel-filtration using Superdex 200 (HiLoad 26/60, 2.6×60 cm, GE Healthcare UK Ltd) at a flow rate of 2.5 ml/min, with PBS as the mobile phase. Fractions containing egg yolk IgG were collected and concentrated with an Amicon Ultra-15 10K (Millipore, Billerica, MA). A portion of the glycoprotein was separated by SDS-PAGE and blotted onto polyvinylidene difluoride (PVDF) membranes for *N*-terminal sequence analysis by Edman degradation, using the Applied Biosystems model 492HT Procise® Protein Sequencer. For matrix assisted laser desorption/ionization-time of flight-mass spectrometry (MALDI-TOF-MS) analysis, an aliquot of each sample (0.5 µl) diluted with distilled water was mixed with 0.5 µl of 10 mg/ml sinapinic acid in 50% acetonitrile and 0.1% trifluoroacetic acid, and analyzed as described previously [16].

### Preparation of Ovomucoid

Ovomucoid was prepared as described previously [17], with some modifications. Briefly, lyophilized egg white (260 mg) was dissolved with 2 ml water. Freshly prepared two volumes of trichloroacetic acid (TCA)/acetone solution (0.5 M TCA : acetone = 1∶2) were slowly added to the egg white, with continuous stirring, and gently mixed overnight at 4°C. After adding 4 ml of cold TCA/acetone solution again and mixing gently, the sample was centrifuged at 3000×g for 25 min at 4°C. To the supernatant, 2.5 volumes of cold acetone was added, with vigorous stirring, and this was kept at 4°C overnight. The precipitate was recovered by centrifugation at 3000×g for 25 min at 4°C, and dissolved in water. After dialyzing against water, recovered ovomucoid was lyophilized.

### Electrophoresis and Western Blotting

Electrophoresis was performed under reducing conditions on a 12.5% SDS-polyacrylamide gel, using egg yolk IgG (1.5 µg/lane), egg white proteins (2.5 µg/lane), or tissue homogenates (20 µg of protein/lane). The separated proteins were transferred to PVDF membranes, followed by detection with Coomassie Brilliant Blue R-250 (CBB) or by antibody/lectin staining, as described previously [15].

### De-*N*-glycosylation of Glycoproteins with Glycoamidase F (GAF)

Glycoproteins were dissolved with PBS containing 1% SDS and denatured by heating at 100°C for 3 min. After cooling to room temperature, the solution was diluted with nine volumes of PBS containing 0.5% Triton X-100, and was incubated with glycoamidase F (GAF, also known as *N*-glycosidase F or PNGase F) at 37°C for 16 h.

### Assays of α/β4GalT(Gal) and β4GalT(GlcNAc)

**Figure 1:**
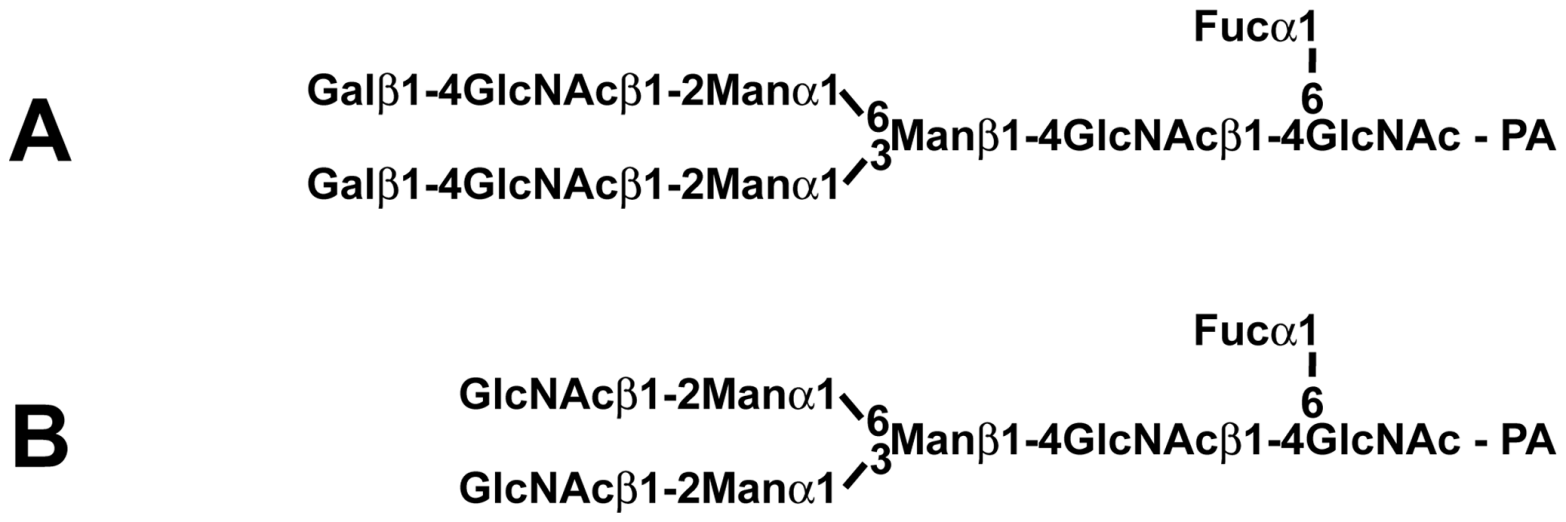
Structures of PA-oligosaccharides used as acceptor substrates for the GalTs assay. PA-derivatized *N*-glycan A and *N*-glycan B were utilized as substrates of α/β4GalTs(Gal) and β4GalT(GlcNAc), respectively.

2-Aminopyridine-(PA)-derivatized substrates A and B (*N*-glycans A and B in Figure 1) were obtained as described previously [15]. Fresh tissues from Japanese quail and Chinese bamboo partridge were kept at −80°C until used, and tissue extracts and microsomal fractions were prepared as described previously [15]. To analyze the activities of GalTs in avian tissues, assays were performed as described in [15]. For general GalT assays, microsomal fractions were used to detect the activity of GalTs. In cases where enzyme activities were not detected in microsomal fractions, the GalT assay was carried out at 37°C for 4 h with 10 mg protein/ml of tissue extract, or for 16 h with 30 mg protein/ml. For the α4GalT(Gal) and β4GalT(Gal) assay, products from PA-substrate A were separated by normal phase HPLC on the Amide-80 column (Tosoh Co., Tokyo, Japan), and then analyzed by reversed-phase HPLC on the ODS column (Shimadzu, Kyoto, Japan) [15]. For the β4GalT(GlcNAc) assay, products from PA-substrate B were analyzed by HPLC on the Amide-80 column.

### Exo-glycosidase Digestion, Permethylation, and MS Analyses for Structural Analysis of *N*-glycans

*N*-Glycans were prepared as described previously [11]. In brief, glycoproteins were reduced and alkylated with dithiothreitol and iodoacetamide, respectively, and digested with trypsin in 50 mM NH_4_HCO_3_ (pH 8.0) at 37°C overnight. After inactivating the enzymes at 100°C for 10 min, oligosaccharides were released with GAF-treatment in 50 mM NH_4_HCO_3_ (pH 8.0) at 37°C overnight. The *N*-glycans were digested with various exo-glycosidases under the following conditions: 2 mU of α2,3-specific neuraminidase from *Macrobdella decora* (Recombinant, Calbiochem) in 20 µl of 50 mM ammonium acetate buffer (pH 6.0), at 37°C for 24 h; 10 mU of α-galactosidase from green coffee bean (Calbiochem) in 20 µl of 50 mM ammonium acetate buffer (pH 6.0), at 37°C for 48 h; and 3 mU of β1,4-galactosidase from *Streptococcus pneumoniae* (Recombinant, Calbiochem) in 20 µl of 50 mM ammonium acetate buffer (pH 6.0), at 37°C for 24 h.

Permethylation of the released *N*-glycans, and subsequent MALDI-MS and MS/MS analyses on a MALDI TOF/TOF (ABI 4700 Proteomic Analyzer) were performed essentially as described previously [18]. Additional nano electrospray ionization (nanoESI)-MS^n^ analyses were performed on an LTQ-Orbitrap XL hybrid FT mass spectrometer (Thermo Scientific). The permethylated glycans in 50% acetonitrile/5 mM sodium acetate were directly infused by static nanoESI. MS^n^ data were each acquired over a period of time and signals were generally averaged from 10 microscans except those of the final MS^4^ stage, which were averaged from 60 microscans or more to give a good signal to noise ratio. The precursor ion isolation widths were set at 3, 5, and 5 mass units for MS^2^, MS^3^, and MS^4^ analysis, respectively, while a 30% normalized collision energy was applied throughout the sequential MS^n^ analyses.

## Results

### Detection of Glycoproteins Containing Galα/β1-4Gal in Avian Egg White Glycoproteins and Egg Yolk IgGs by Western Blot Analysis

**Figure 2:**
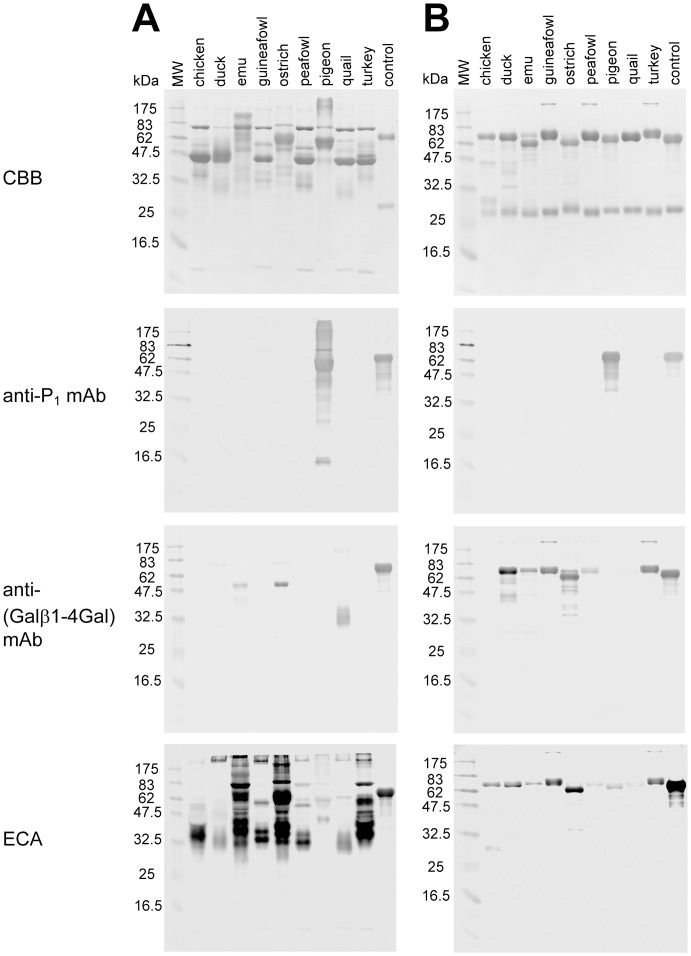
Antibody/lectin-staining of avian egg white glycoproteins and isolated egg yolk IgG. Egg white glycoproteins (A, 2.5 µg/lane) or egg yolk IgG (B, 1.5 µg/lane) from chicken, duck, emu, guineafowl, ostrich, peafowl, pigeon, quail, and turkey were blotted onto a membrane, and visualized with CBB-staining. Pigeon IgG (for CBB and anti-P_1_ mAb stainings) and α-galactosidase-treated pigeon IgG (for anti-(Galβ1-4Gal) mAb and ECA stainings) were used as controls [11].

**Figure 3:**
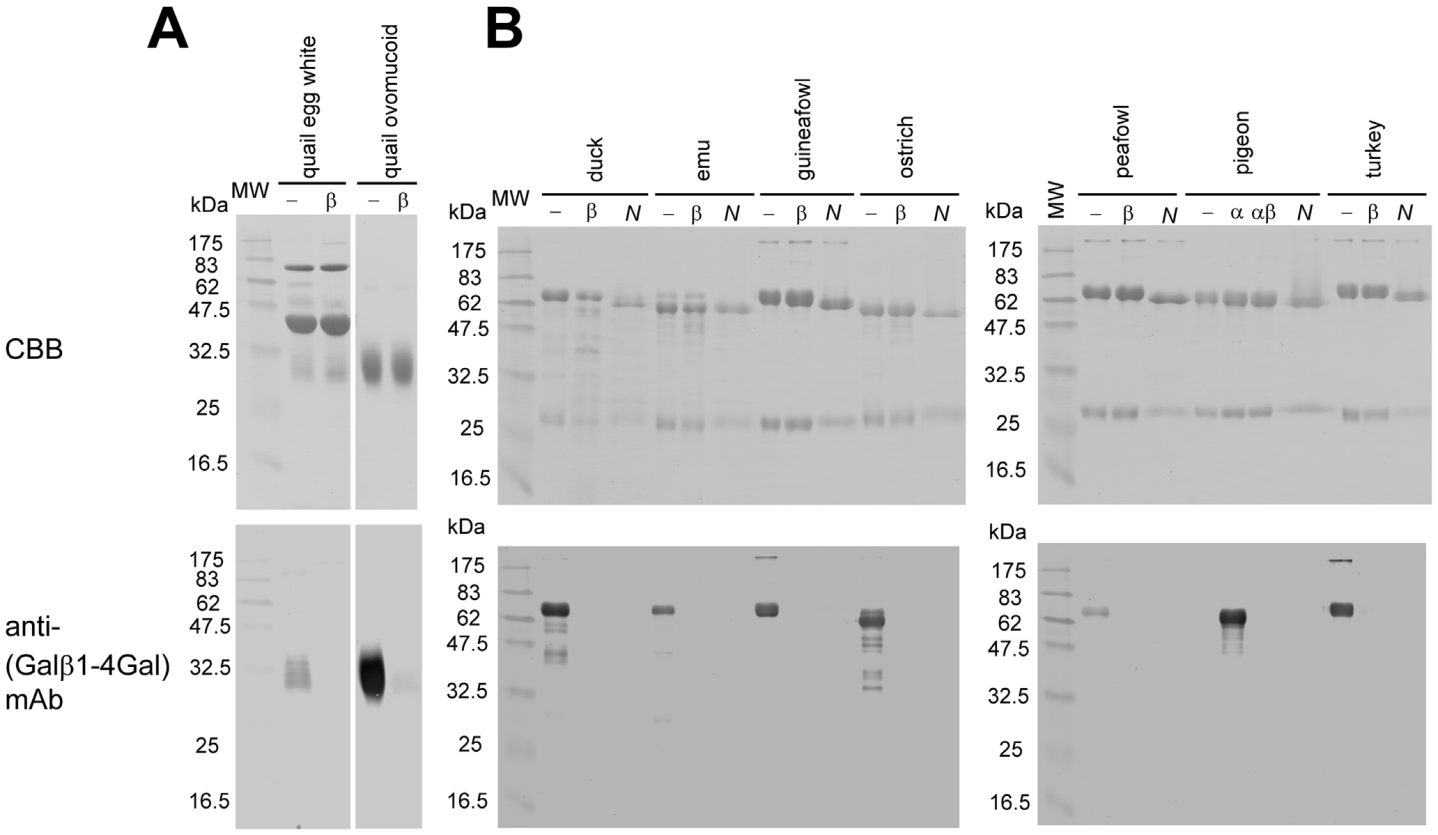
Digestion of quail ovomucoid and avian egg yolk IgG with exogalactosidases or glycoamidase F (GAF). *A*, Quail egg white proteins and quail ovomucoid were untreated (−) or treated with β4-galactosidase (β). *B*, The isolated egg yolk IgGs from duck, emu, guineafowl, ostrich, peafowl, and turkey were untreated (−) or treated with β4-galactosidase (β) or GAF (*N*). In *B*, pigeon egg yolk IgG was treated with α-galactosidase (α), α-galactosidase and then β4-galactosidase (αβ), or GAF (*N*). Each sample was separated by SDS-PAGE, transferred onto PVDF membranes, and stained with CBB or anti-(Galβ1-4Gal) mAb.

To determine whether Galα1-4Gal and Galβ1-4Gal epitopes are present on avian glycoproteins, egg white proteins from ostrich, emu, quail, chicken, peafowl, turkey, guineafowl, duck, and pigeon (Table 1) were analyzed by SDS-PAGE and western blotting. The major glycoproteins in egg whites were visualized with Coomassie Brilliant Blue R-250 (CBB)-staining (Figure 2A). Egg white glycoproteins from all of the species listed in Figure 2A, were stained with *Erythrina cristagalli* agglutinin (ECA), which recognizes Galβ1-4GlcNAc. This fact suggests that all species tested have the substrates for α/β4GalTs(Gal) in the cells that biosynthesize the glycoproteins. In contrast, egg white glycoproteins from all species except pigeon did not stain with anti-P_1_ mAb, which recognizes Galα1-4Galβ1-4GlcNAc (Figure 2A). This result is consistent with the previous observations that Galα1-4Gal is absent in egg whites from Ratitae (ostrich and emu) and Galloanserae (quail, chicken, peafowl, turkey, guineafowl, and duck) [7]. Most of the egg white glycoproteins, which were visualized by staining with CBB and/or ECA, did not stain with anti-(Galβ1-4Gal) mAb. However, some bands of glycoproteins of emu, ostrich, and quail clearly were visualized by staining with this antibody (Figure 2A). As we have demonstrated previously, ostrich expresses β4GalT(Gal) in various tissues [15]. Because emu is a close relative of ostrich and belongs to the same order, Struthioniformes (Table 1), the ability to express Galβ1-4Gal epitopes on glycoproteins is most likely conserved in both ostrich and emu. In contrast, since quail is not close to ostrich nor pigeon, but close to chicken (Table 1), the presence of broad bands of around 30–34 kDa in the egg white of quail stained with anti-(Galβ1-4Gal) mAb (Figure 2A) was not expected. According to the molecular size detected by SDS-PAGE, the protein was most likely ovomucoid. We confirmed that it was ovomucoid by isolating this glycoprotein from the egg white using the trichloroacetic acid (TCA)-precipitation method as described previously [17]. As shown in Figure 3A, the isolated quail ovomucoid and the corresponding glycoproteins in egg white clearly stained with the anti-(Galβ1-4Gal) mAb, and no longer stained with the antibody after β4-galactosidase-digestion. Accordingly, the results of immunostaining of avian egg white glycoproteins indicated the possibility that at least emu, ostrich, and quail express glycoproteins containing Galβ1-4Gal epitopes.

**Table 1:** List of birds whose eggs and/or tissues were used.

(Infraclass/Parvclass)Order Family^a^	Common name	Latin name
(Ratitae)StruthioniformesStruthionidae	Ostrich	**Struthio camelus**
Casuariidae	Emu	*Dromaius novaehollandiae*
(Galloanserae)GalliformesPhasianidae	Japanese Quail	*Coturnix japonica*
	Chinese BambooPartridge	*Bambusicola thoracicus*
	Chicken (Red Junglefowl^b^)	*Gallus gallus*
	Indian Peafowl	*Pavo cristaus*
	Wild Turkey	*Meleagris gallopavp*
Numididae	Helmet Guineafowl	*Numida meleagris*
AnseriformesAnatidae	Duck (Mallard^c^)	*Anas platyrhynchos*
(Neoaves)ColumbiformesColumbidae	Pigeon (Rock Dove)	*Columba livia*

a Based on the DNA-DNA hybridization method by Sibley, *et al* [9].

b Red Junglefowl is believed to be the direct ancestor of the domestic chicken.

c Mallard is believed to be the ancestor of domestic ducks.

We previously found that the Galβ1-4Gal epitope is abundant in *N*-glycans from pigeon IgG [11], but not in those from pigeon egg white glycoproteins [12], [13]. Since avian IgG, either in serum or egg yolk, is produced in antibody-producing cells, the glycan structures are possibly different from those produced in the oviduct. Thus, we also analyzed egg yolk IgGs from the nine avian species. The identities of the isolated IgGs were confirmed by determining the *N*-terminal sequences of their heavy chains, and their molecular masses, by MALDI-TOF-MS (Table 2) and SDS-PAGE (Figure 2B). The heavy chains of IgG from all nine species were stained with ECA, although those from peafowl and quail were stained relatively weakly (Figure 2B). Only pigeon IgG-heavy chain was stained with anti-P_1_ mAb, confirming the presence of Galα1-4Gal on pigeon glycoproteins and the absence of Galα1-4Gal on glycoproteins from Ratitae and Galloanserae [7]. In contrast, anti-(Galβ1-4Gal) mAb stained IgG-heavy chains from duck, emu, guineafowl, ostrich, peafowl, and turkey, but not from chicken, pigeon, and quail (Figure 2B). The bands stained with anti-(Galβ1-4Gal) mAb were no longer stained after treatment with β4-galactosidase or GAF (Figure 3B), suggesting that heavy chains from duck, emu, guineafowl, ostrich, peafowl, and turkey IgGs possess *N*-glycans containing Galβ1-4Gal. Among IgG-heavy chains from the rest of three species, i.e., pigeon, chicken, and quail, which were not stained with anti-(Galβ1-4Gal) mAb (Figure 2B), *N*-glycan structures of pigeon IgG-heavy chains possess Galα1-4Galβ1-4Galβ1-4GlcNAc sequences [11]. Since the majority of Galβ1-4Gal epitopes on the IgG-heavy chains is masked with α4-galactosylation, Galβ1-4Gal epitope was detected by anti-(Galβ1-4Gal) mAb-staining only after an α-galactosidase-digestion (Figure 3B). The newly exposed Galβ1-4Gal on pigeon IgG was removed by the following treatment with β4-galactosidase. In contrast, the heavy chains of chicken and quail IgG were not stained with anti-(Galβ1-4Gal) mAb, because Galβ1-4Gal epitopes are absent in *N*-glycans of both chicken [16], [19] and quail [20] IgG as reported previously.

**Table 2:** Identification of avian egg yolk IgG^a^.

Species	[M+H]^+^	*N*-terminal amino acid sequence(heavy chain)
chicken	170304.4	AVTLDESGGGLQT
duck	169784.6	AATLDESGGGLV
emu (upper band)^b^	169912.4	AVPLSESGGG
emu (lower band) b	163678.5	AVQLSESGGGLQPPG
guineafowl	168832.0	AVTLDESGGGLQ
ostrich	169857.6	AVPLVESGGGLQ
peafowl	168035.6	AVTLDESGGGLQAPG
pigeon	171870.4	AIELVESGGGLVSPG
quail	173317.6	AVTLDETGGGLYAPG
turkey	171794.4	AVTLDESGGGLQT

a Molecular masses of whole isolated avian IgGs, also called IgYs, were approximately 170 kDa, and are larger than those of mammals (approximately 150 kDa).

b Emu IgG showed two bands of around 65 kDa in SDS-PAGE at reducing conditions, with CBB-staining (Figure 2B). The higher band shifted to the lower band by treatment with glycoamidase F (Figure 3B), suggesting that the different molecular sizes are mainly due to differences in glycosylation.

### Structural Analysis of *N*-glycans of Egg Yolk IgG from Turkey, Guineafowl, and Peafowl

**Figure 4:**
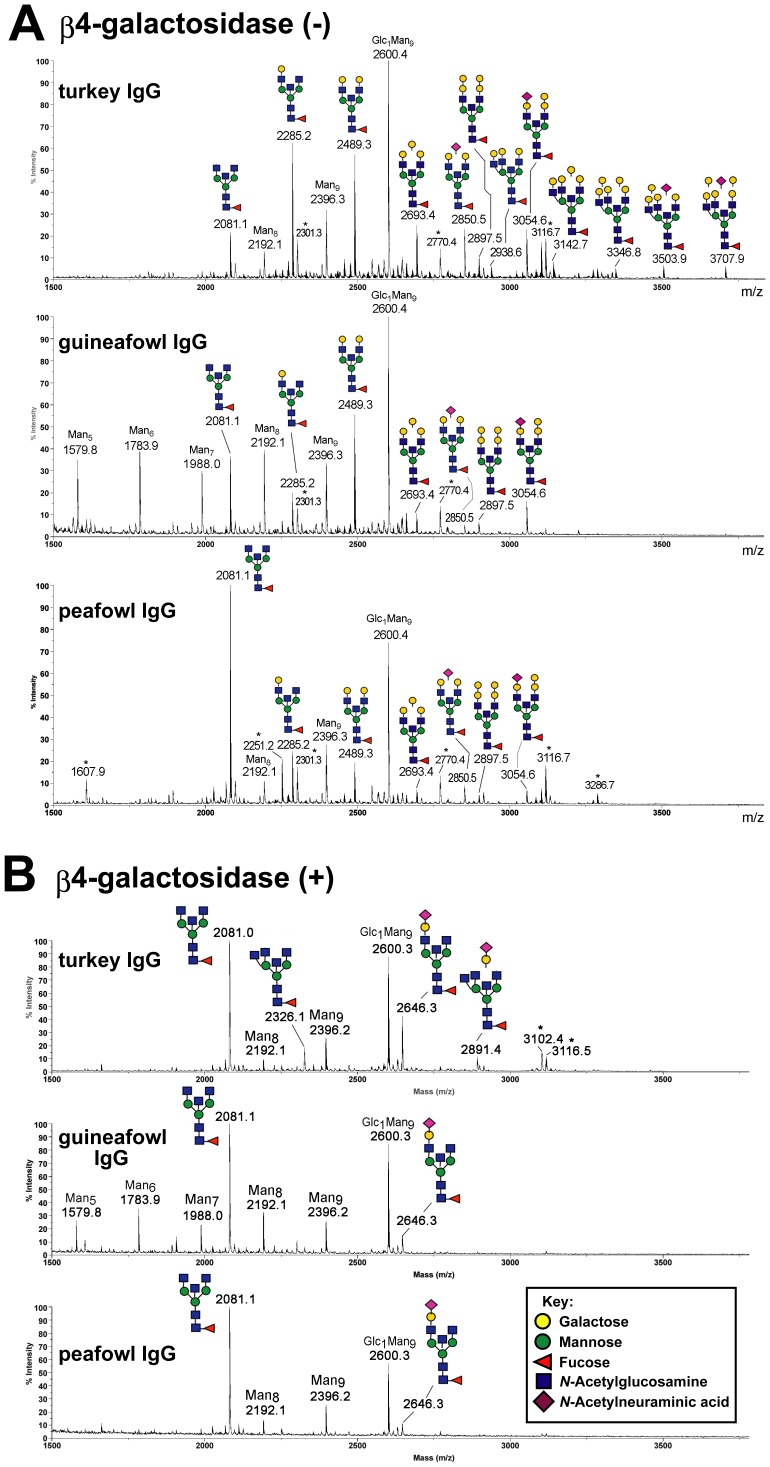
MALDI-MS profiles of permethylated total IgG *N*-glycans from turkey, guineafowl, and peafowl. The avian IgG *N*-glycans before (A) and after (B) β4-galactosidase digestion were permethylated and analyzed with MALDI-MS. The most probable structures corresponding to the afforded major [M+Na]^+^ molecular ion signals are annotated along with their monoisotopic values. Assignments are based on a combination of inferred glycosyl compositions, susceptibility to β-galactosidase digestion (B) and MS/MS data obtained on the more abundant components (Figure 5). The MS/MS data positively identify *N*-glycans with a bisecting GlcNAc as the major complex type structures but do not rule out the presence of alternative non-bisected structures. Unassigned signals that did not give interpretable glycan-like MS/MS spectra are marked with an asterisk (*). Monosaccharide symbols used conform to the recommendations by the Consortium for Functional Glycomics.

**Figure 5:**
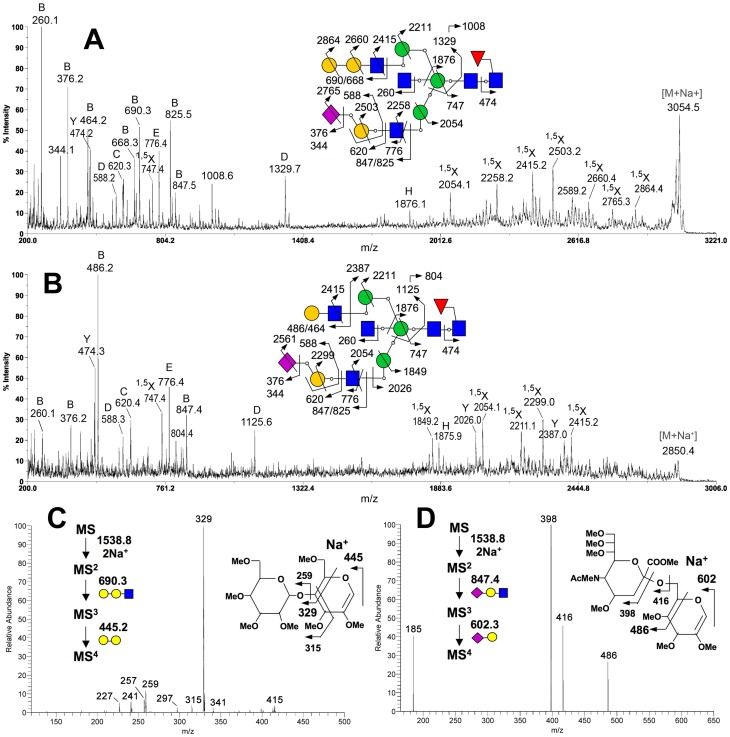
MALDI MS/MS and nanoESI-MS^n^ analysis of the permethylated *N*-glycans of avian IgG. High energy CID MS/MS analyses on MALDI-TOF/TOF readily identified the presence of bisecting GlcNAc and core fucosylation on most complex type structures, as shown by the representative spectra for the sodiated molecular ions at *m/z* 3054 (A) and 2850 (B) from permethylated turkey and peafowl IgG *N*-glycans, respectively. The presence of Gal-Gal-GlcNAc in the former but not the latter is confirmed by the non-reducing terminal B ion at *m/z* 690. The B ion at *m/z* 464 in (A), which corresponds to the non-reducing terminal oxonium ion of Hex-HexNAc, indicates that an alternative non-bisected, triantennary structural isomer was also present but at smaller amount since no other supporting ions could be detected. The linkage of the Gal-Gal was established by nanoESI-MS^n^ analysis, by observing the characteristic ^3,5^A ion at *m/z* 329 at the level of MS^4^ (C). Sialylation at 6 and not 3 position of Gal was likewise established by detecting the characteristic ^3,5^A ion at *m/z* 486 at the level of MS^4^ (D). Assignments of all other major fragment ions are schematically illustrated on each of the Figures, adopting the ion nomenclature as described previously [18], [21].

To confirm the presence of Galβ1-4Gal in egg yolk IgGs from turkey, guineafowl, and peafowl, which belong to Galliformes as chicken, permethylated *N*-glycans from these IgGs were analyzed by MS and MS/MS analyses (Figure 4 and 5). The glycosyl composition of the major peaks detected can be readily assigned, from which tentative structures can be inferred as annotated in Figure 4A. Candidate structures carrying Galβ-Gal-GlcNAc termini were confirmed by a combination of α- and β-galactosidase digestions and MS/MS analyses, which also support the presence of bisecting GlcNAc and core fucosylation on most of the identified components. Importantly, while α-galactosidase treatment did not affect the resulting MS profiles of the permethylated glycans, β-galactosidase digestion trimmed all antenna of complex type *N*-glycans to single GlcNAc, except those protected by sialylation (Figure 4B). The data therefore not only indicate that all Gal-Gal are of β-linkage but also that sialylation does not occur on the Galβ-Galβ-GlcNAc epitope. This is consistent with the fragment ions afforded by high energy CID MS/MS analysis on a MALDI TOF/TOF, which corroborated the presence of non-reducing terminal NeuAc-Hex-HexNAc, Hex-Hex-HexNAc, Hex-HexNAc and HexNAc, but not NeuAc(Hex-Hex-HexNAc).

The representative MALDI MS/MS spectra acquired on the monosialylated complex type *N*-glycans with and without the Gal-Gal-GlcNAc epitope are shown in Figure 5A and B, respectively. In general, the structures assigned are well supported by the expected series of reducing and non-reducing terminal ions [18], [21], as illustrated and annotated. The ring cleavage ^1,5^X ions, in particular, afforded complete sequencing from the non-reducing end (e.g., *m/z* 2864, 2660, 2415 at the Hex-Hex-HexNAc sequence shown in Figure 5A), supplemented by the Y_1_ ion at *m/z* 474, which unambiguously localized the single Fuc to reducing end GlcNAc. The D ions formed at the β-Man (*m/z* 1329 in Figure 5A and *m/z* 1125 in Figure 5B) indicated that the sialylated antennae are preferentially located at the 3-arm. Each was accompanied by an ion corresponding to loss of 321 units, which have previously been noted as indicative of the presence of bisecting GlcNAc [21], [22]. This is further supported by the common H ion (*m/z* 1876 in Figure 5A and 5B) formed via concerted elimination of the entire 6-arm substituents and the bisecting GlcNAc from the β-Man.

The Gal-Gal-GlcNAc epitope, where present, was additionally identified by the characteristic sodiated B ion at *m/z* 690. This ion was also afforded by nanoESI-MS^2^ analysis on the doubly sodiated molecular ion, which could be further isolated for MS^3^ to induce the formation of a sodiated B ion at *m/z* 445, corresponding to Hex-Hex (Figure 5C). Upon MS^4^ analysis, the detection of a single major ^3,5^A_1_ ring cleavage ion at *m/z* 329 fully supported a Gal-4Gal linkage, as it cannot be formed from the alternative Gal-3Gal epitope [23]. The linkage for the NeuAc-Gal was first inferred to be α2-6 from high energy CID MALDI MS/MS analysis (Figure 5A, B) by virtue of detecting the characteristic D ion at *m/z* 588, along with the absence of a dominant peak at *m/z* 356 indicative of α2-3 linkage [24], [25]. Similar to the nanoESI-MS^n^ analysis of Gal-Gal-GlcNAc, the sodiated B ion for NeuAc-Gal-GlcNAc produced from MS^2^ of the doubly sodiated parent could likewise be isolated for MS^3^ to give a sodiated B ion at *m/z* 602 corresponding to NeuAc-Hex, which can then be further isolated for MS^4^ to produce a ^3,5^A_1_ ring cleavage ion at *m/z* 486, supportive of the NeuAc2-6Gal linkage (Figure 5D). Finally, the predominance of α2-6 sialylation is consistent with the *N*-glycan samples being largely resistant to α2-3 specific sialidase digestion.

Taken together, it can be concluded that *N*-glycans from turkey, guineafowl, and peafowl IgGs comprise a population of complex type *N*-glycans that carry the Galβ1-4Galβ1-4GlcNAc sequence. In addition, they share several structural features commonly found in other avian IgG *N*-glycans reported thus far [11], [16], [19], [20], [21]. First, complex-type *N*-glycans from the avian IgGs are mainly limited to biantennary and triantennary structures in size, and the majority of these complex-type structures are bisected and core fucosylated. Biantennary structures were mainly detected for complex-type *N*-glycans of IgGs from guineafowl and peafowl, whereas there was additional heterogeneity in those of turkey IgG due to the presence of triantennary structures, such as *m/z* 2938.6, 3142.7, 3346.8, 3503.9, and 3707.9 (Figure 4A). Otherwise, the MS profiles of complex-type *N*-glycans afforded by the three samples were fairly similar. Second, the β-Gal capping is incomplete with many of the LacNAc termini remain non-capped or Neu5Acα2-6-sialylated. Third, the three avian IgG *N*-glycans comprise a significant amount of high mannose structures including a Hex_10_HexNAc_2_ structure, most likely corresponding to the Glc_1_Man_9_GlcNAc_2_ structure commonly found in avian IgG, such as chicken [16], [19], quail [20], pigeon [11], and gull [21]. Notably, Glc_0–1_Man_8–9_GlcNAc_2_ were the only major high mannose-type *N*-glycans detected for IgGs from turkey and peafowl (Figure 4A), whereas the IgG from guineafowl appears to carry more of the smaller Man_5–7_GlcNAc_2_ structures.

### Analysis of GalTs Activities and Immunostaining for Galα/β1-4Gal in Quail and Bamboo Partridge Tissues

The detection of Galβ1-4Gal in quail ovomucoid (Figure 2A, 3A) was unexpected, because the reported major *N*-glycan structures of quail ovomucoid did not contain Galβ1-4Gal [26]. Moreover, quail egg yolk IgG did not stain with anti-(Galβ1-4Gal) mAb, unlike those of other species in Galloanserae other than chicken (Figure 2B). Thus, to confirm the expression of Galβ1-4Gal in quail, we examined the presence of Galβ1-4Gal in various tissues of quail by detecting β4GalT(Gal) activities and by immunoblotting. Furthermore, although quail, peafowl, and chicken belong to the same family, Phasianidae (Table 1), we previously determined that Galβ1-4Gal is absent in chicken [15]. To clarify whether the absence of Galβ1-4Gal is specific to chicken among the Phasianidae, we also screened for Galβ1-4Gal in bamboo partridge, which is proposed to be the closest lineage to the genus *Gallus*, which includes chicken, based on molecular phylogenetic analyses [27], [28], [29], [30].

**Figure 6:**
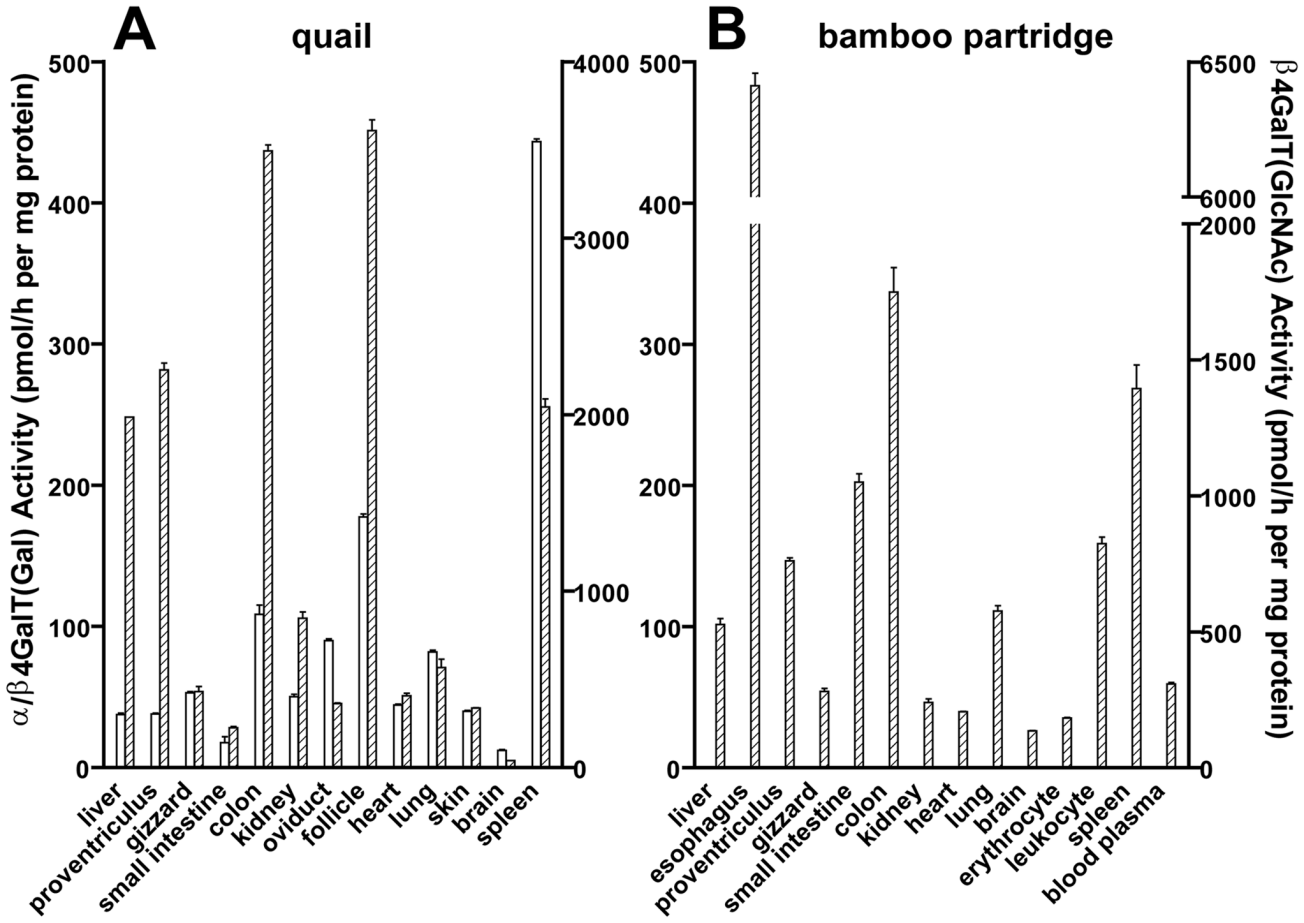
Analysis of the tissue distributions of α4GalT(Gal), β4GalT(Gal), and β4GalT(GlcNAc) from quail and bamboo partridge. Microsomal fractions or tissue extracts were prepared from various tissues of quail (A) or bamboo partridge (B). The values represent the means ±S.D. of duplicate samples. Scales for β4GalT(Gal) (*open bar*) activities were indicated on the left *y*-axes, and those for β4GalT(GlcNAc) (*hatched bar*) activities on the right *y*-axes of each graph. No α4GalT(Gal) activities were detected in any tissues from quail or bamboo partridge. β4GalT(Gal) activities were detected only in tissues from quail.

Both the α/β4GalTs(Gal) assay and the β4GalT(GlcNAc) assay were performed on tissues from quail and bamboo partridge by the same method described previously, using the 2-aminopyridine (PA)-derivatized *N*-glycans (Figure 1) as acceptor substrates. In quail, β4GalT(Gal) and β4GalT(GlcNAc) activities were detected in all tissues (Figure 6A), although the activities in the small intestine and brain were relatively low. For other tissues, microsomal fractions were used as enzyme sources. No α4GalT(Gal) activity was detected in any tissue from quail, consistent with our assumption that Galα1-4Gal is absent from Galloanserae. In contrast, all tissues from bamboo partridge revealed β4GalT(GlcNAc) activities, but not α/β4GalT(Gal) activities (Figure 6B). Even when the concentration of proteins in the tissue extracts was increased up to 30 mg/ml and the reaction mixtures were incubated for longer periods (∼16 h) at 37°C, no products were detected with α/β4GalT(Gal).

**Figure 7:**
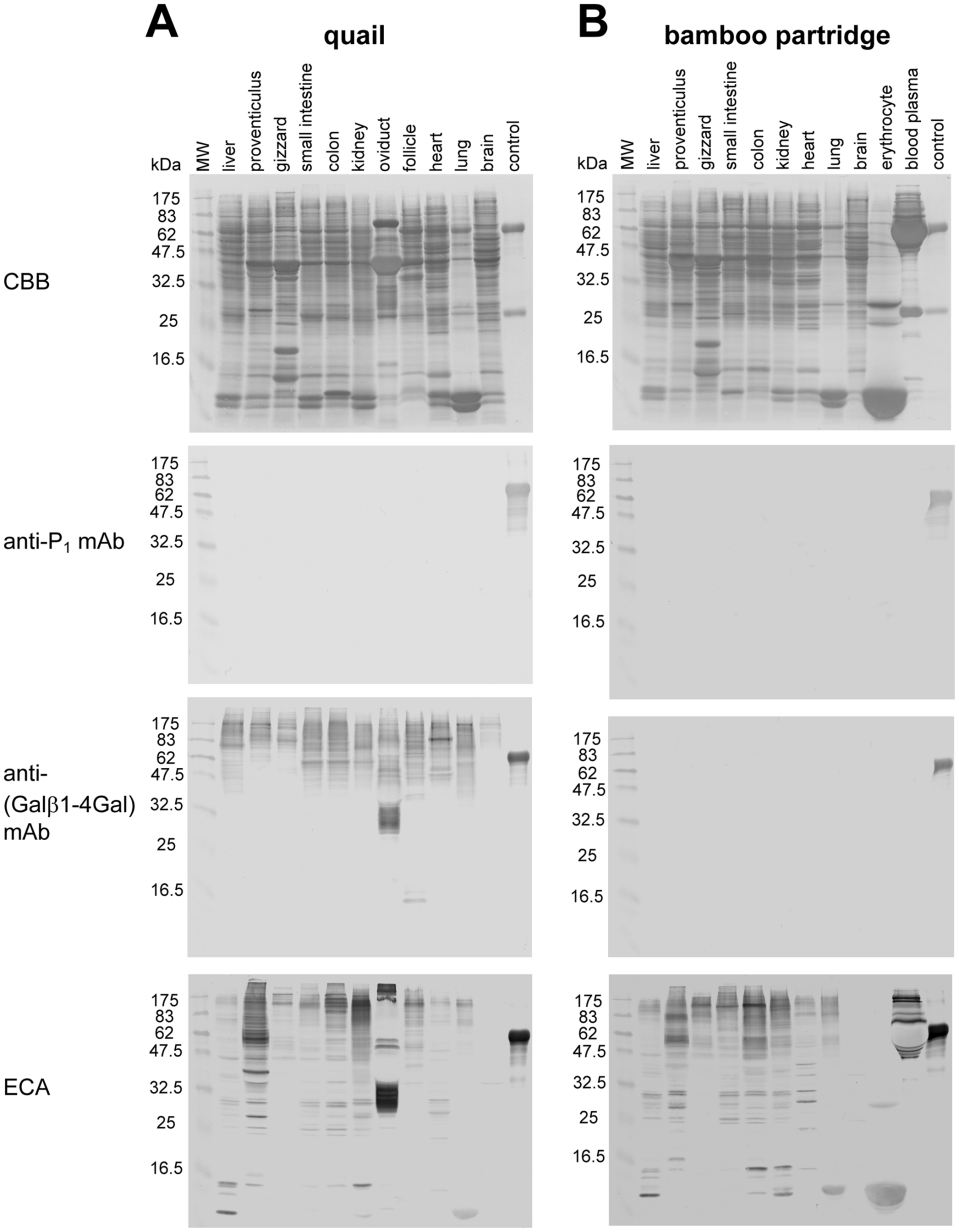
Antibody/lectin-staining of protein extracts from various tissues of quail (A) and bamboo partridge (B). Proteins (20 µg/lane) blotted onto PVDF membranes were visualized with CBB-staining. Pigeon IgG (for CBB and anti-P_1_ mAb stainings) and α-galactosidase-treated pigeon IgG (for anti-(Galβ1-4Gal) mAb and ECA stainings) were used as controls.

The results of antibody/lectin-staining for tissues from quail and bamboo partridge (Figure 7) were correlated with their expression of α/β4GalTs(Gal) and β4GalT(GlcNAc). Glycoproteins containing the Galβ1-4Gal epitope were expressed widely in the body of quail, while glycoproteins with the Galβ1-4Gal epitope were absent in bamboo partridge, as we previously demonstrated in chicken [15].

## Discussion

**Figure 8:**
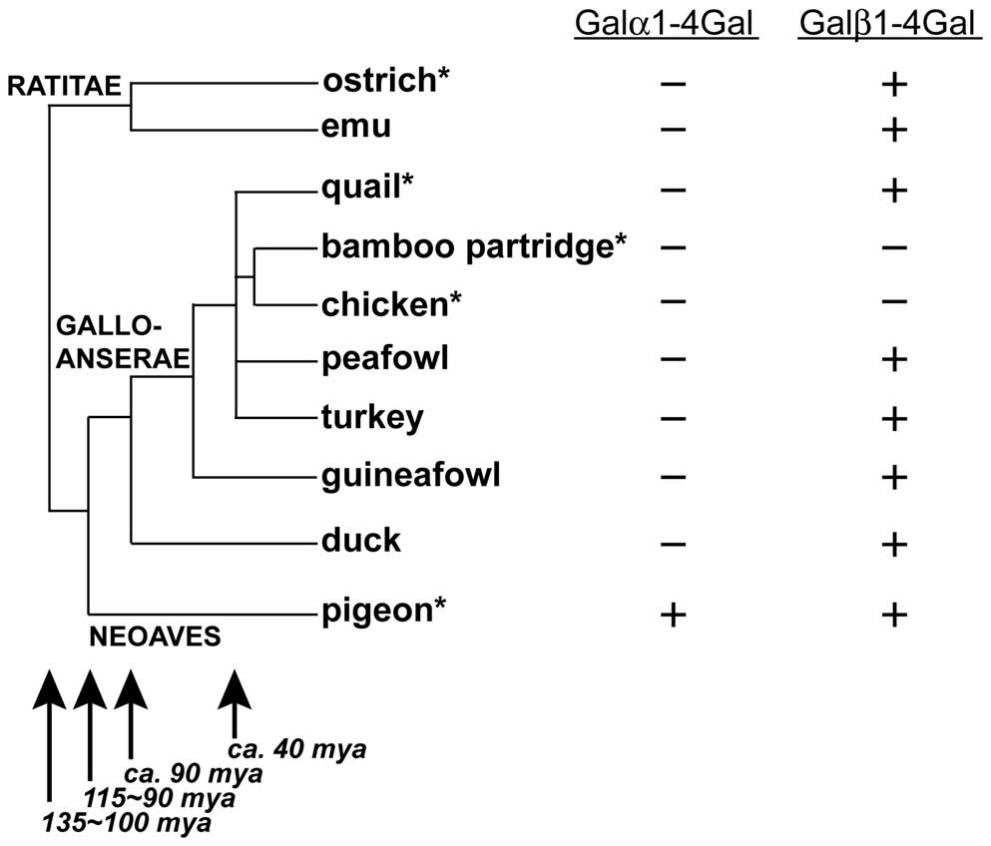
Phylogenetic relationship of birds used in this study. The phylogenetic tree is based on documented literature [27], [28], [29], [30], [32], [34], but the relationships within Phasianidae (quail, bamboo partridge, chicken, peafowl, and turkey) were simplified due to the lack of consensus among proposed phylogeny. The presence (+) and absence (−) of Galα1-4Gal or Galβ1-4Gal on glycoproteins are based on detections in various tissues from species indicated with an *asterisk* (*) or on detections on egg while/yolk glycoproteins from all species except bamboo partridge. mya, million years ago.

Birds are one of the higher vertebrates that radiated in the Tertiary period (65–1.6 million years ago (mya)) together with mammals. However, the glycan aspects of birds might have been evolutionarily differentiated from those of mammals, after the ancestors of birds and mammals separated about 310 mya [31]. Modern birds (Neornithes) are monophyletic, and more than 9,000 avian species were identified in the world. They classified into three large taxa, namely Ratitae, Galloanserae, and Neoaves (Figure 8) [9], [32], [33], . It has been proposed that Ratitae was the first to diverge from the others, parting 135–100 mya, and that Galloanserae and Neoaves separated 115–90 mya [35]. Neoaves is the largest group, containing about 95% of modern birds species, whereas Ratitae and Galloanserae are rather small groups, consisting of <1% and <5% of modern bird species, respectively.

Galβ1-4Gal in birds was initially identified in *O*-glycans from Chinese swiftlet [10] and in *N*-glycans of IgG from pigeon [11]. Since both species belong to Neoaves, Galβ1-4Gal seems to be expressed in some other birds belonging to Neoaves. The expression of Galβ1-4Gal in Neoaves is supported by the fact that another Neoaves, zebra finch, possesses a gene which is similar to pigeon β4GalT(Gal) cDNA [36]. In addition, we found that Galβ1-4Gal on glycoproteins is also expressed in ostrich, which belongs to Ratitae [15]. As shown in this study, another bird in Ratitae, emu, was also found to express Galβ1-4Gal, at least on egg yolk IgG (Figure 2B, 3B), supporting the presence of Galβ1-4Gal in Ratitae. In contrast, there were no reports that birds in Galloanserae express Galβ1-4Gal on glycoproteins previously.

Galloanserae is a smaller group of avians in terms of the number of species comparing to those of Neoaves. However, some of the species belonging to Galloanserae are very familiar. The chicken, for example, was domesticated more than 8,000 years ago [37]. The two major avian orders in Galloanserae are Galliformes (e.g., quail, bamboo partridge, chicken, peafowl, turkey, guineafowl) and Anseriformes (e.g., duck), which diverged around 90 mya [35], [38], [39], [40]. The phylogenetic relationship among avian species used in this study and the expression of Galα/β1-4Gal is summarized in Figure 8. No activities of α/β4GalTs(Gal) were detected in chicken and bamboo partridge, while all other species analyzed in this study did express Galβ1-4Gal. Considering that Galβ1-4Gal is expressed in a wide range of modern birds, including several species phylogenetically close to chicken, it is most likely that the ability to express Galβ1-4Gal is conserved among avian species, but was lost in the ancestors of chicken and bamboo partridge after they separated from quail, peafowl, and turkey (around 40 mya [35]), or from their ancestors. Due to a lack of information, it has not been recognized that structural features of the glycans expressed in chicken are rather exceptional among those of avian species.

The presence or absence of Galβ1-4Gal in pigeon, ostrich, quail, chicken, and bamboo partridge were correlated with the activities of β4GalT(Gal) detected in various tissues (Figure 6, 7) [15]. Since no β4GalT(Gal) activity was detected in either chicken or bamboo partridge, this enzyme is inactive or not expressed in these birds. This possibility is supported by the fact that the genes which are similar to the pigeon β4GalT(Gal) cDNA are absent in chicken genome [36]. Currently (2012, November), the whole genome sequence of turkey is also available in the database of National Center for Biotechnology Information (NCBI), as the third avian species, following those of chicken and zebra finch. We found the gene similar to the pigeon β4GalT(Gal) cDNA in the turkey genome, supporting our results that Galβ1-4Gal epitope is also expressed in this bird (Figure 2, 3, 4, 5). Moreover, genes similar to the pigeon β4GalT(Gal) cDNA are also found in zebrafish (*Danio rerio*), African clawed frog (*Xenopus laevis*), and Western clawed frog (*X. (Silurana) tropicalis*) [36], as well as in green anole (*Anolis carolinensis*), a reptile. Although the presence of Galβ1-4Gal epitope in the reptiles remains to be clarified, we confirmed the presence of Galβ1-4Gal epitope in zebrafish and African clawed frog by the western blot analysis [14]. If the Galβ1-4Gal epitope is also expressed in reptiles, it is possible to consider that the ability to express this epitope might be inherited from fish, amphibians, and/or reptiles to birds, but not to mammals.

The expression of Galβ1-4Gal among birds and the loss of expression in chicken are reminiscent of the species-specific expression of Galα1-3Gal [4], [41] and *N*-glycolylneuraminic acid (NeuGc) [42], [43] in most mammals. Expression of Galα1-3Gal was lost in humans, apes, and Old World monkeys, and NeuGc is absent specifically in humans, among all the mammals. In both cases, the expressions were abolished by the inactivation of genes encoding critical enzymes for the biosynthesis of these glycans. Since human, apes, and Old World monkeys (catarrhine primates), which lost the active α3GalT(Gal) genes, are known to produce high titers of natural antibodies against Galα1-3Gal structures [4], it is speculated that loss of the active α3GalT(Gal) is advantageous for these species to protect themselves against microbes or viruses expressing Galα1-3Gal epitopes [5]. In addition, recent reports of animal models, in which the expression of these glycans was genetically disrupted, revealed the development of some abnormalities, such as cataracts in (Galα1-3Gal)-deficient mice [44], and a diminished acoustic startle response in NeuGc-deficient mice [45]. These observations suggest that the glycans are not indispensable for ontogenesis of mice, but confer an advantage in maintaining normal physiologic homeostasis. Koike *et al.* (2007) reported that there is strong purifying selection for preserving the gene encoding α3GalT(Gal) in noncatarrhine mammals, and proposed that loss of the active α3GalT(Gal) gene in catarrhines became possible only after alternative and/or more beneficial glycosyltransferase activity evolved in the ancestors of catarrhines [46]. Some advantages may also exist to conserve the expression of Galβ1-4Gal in birds, as suggested in the case of Galα1-3Gal and NeuGc in mammals.

The absence of Galβ1-4Gal can also provide some advantages in excluding foreign organisms that express this epitope. It is reported that the natural antibody against Galβ1-4Gal is rich in human [47], which do not express Galβ1-4Gal epitopes. Chicken and bamboo partridge are potentially capable of producing the antibodies against the carbohydrate antigens containing Galβ1-4Gal, if they also do not express Galβ1-4Gal on glycolipids. The expression of Galβ1-4Gal on glycolipids among birds remains unknown, because we used only *N*-glycans as acceptor substrates to detect activities of GalTs in this study. There may or may not be other β4GalTs(Gal) which act only for glycolipids.

Finally, our findings are also useful to study pathogens of birds. The presence of unique glycan epitopes of avian species was not well focused on so far. One of the reasons is that the glycan aspects from chickens and humans are somehow similar, i.e., the absence of Galα1-4Gal, Galβ1-4Gal, Galα1-3Gal [7], [41], and NeuGc [48], [49], [50] on glycoproteins. However, as we have indicated in this study, glycan profiles were differentiated during the history of birds and mammals, and the similarities between chicken and human seem to be coincidental. Because Galβ1-4Gal is widely distributed among birds, there may be some microbes or toxins that recognize this glycan epitope for cell surface attachments. The different glycan expression patterns among birds result in different susceptibilities to certain infectious diseases and contribute to the prevention of interspecies transmission. Since the avian species such as chicken, turkey, and duck are very important sources for our diet, studies on the molecular basis of avian infectious diseases is necessary for poultry breeding and for the stable supply to markets.
